# Validity of hypertensive disorders of pregnancy diagnoses in the Swedish pregnancy register using a contemporary cohort

**DOI:** 10.1111/aogs.70286

**Published:** 2026-06-12

**Authors:** Mark Frick, Lina Bergman, Ylva Carlsson, Henrik Imberg, Peter Lindgren, Peter Conner, Stefan R. Hansson, Marius Kublickas, Anders Larsson, Sara Hellström, Anna‐Karin Wikström, Anna Sandström

**Affiliations:** ^1^ Department of Women's Health, Division of Gynecology and Reproductive Medicine Karolinska University Hospital Stockholm Sweden; ^2^ Department of Obstetrics and Gynecology Sahlgrenska University Hospital Gothenburg Sweden; ^3^ Department of Obstetrics and Gynecology, Institute of Clinical Sciences University of Gothenburg Gothenburg Sweden; ^4^ Department of Obstetrics and Gynecology Stellenbosch University South Africa; ^5^ Department of Women's and Children's Health Uppsala University Uppsala Sweden; ^6^ Centre of Perinatal Medicine and Health, Institute of Clinical Sciences University of Gothenburg Gothenburg Sweden; ^7^ Department of Women's and Children's Health Karolinska Institute Stockholm Sweden; ^8^ Center for Fetal Medicine Karolinska University Hospital Stockholm Sweden; ^9^ Department of Obstetrics and Gynecology Institute of Clinical Sciences, Lund, Lund University and Skåne University Hospital Lund/Malmö Sweden; ^10^ Department of Medical Sciences, Clinical Chemistry Uppsala University Uppsala Sweden; ^11^ Department of Pediatrics, Institute of Clinical Sciences University of Gothenburg Gothenburg Sweden; ^12^ Department of Neonatology, Queen Silvia Children's Hospital Sahlgrenska University Hospital Gothenburg Sweden; ^13^ Department of Women's Health, Division of Obstetrics Karolinska University Hospital Stockholm Sweden; ^14^ Clinical Epidemiology Division, Department of Medicine, Solna Karolinska Institute Stockholm Sweden

**Keywords:** hypertensive disorders of pregnancy, medical record, preeclampsia, Swedish pregnancy register, validation

## Abstract

**Introduction:**

The validity of hypertensive disorders of pregnancy (HDP), including preeclampsia, gestational hypertension, and chronic hypertension, is essential for both clinical management and research. The Swedish Pregnancy Register automatically retrieves diagnostic codes based on the Swedish version of the International Classification of Diseases 10th revision (ICD‐10), as recorded in antenatal, obstetric, and neonatal electronic medical records. The aim of the study was, for the first time, to assess the validity of HDP diagnoses within the Swedish Pregnancy Register in a contemporary population.

**Material and Methods:**

A multicenter validation study of women with deliveries from 2021 to 2023 who participated in the prospective Swedish study for Improving Maternal Pregnancy And Child ouTcomes (IMPACT) cohort. Women with a HDP diagnosis in the Swedish Pregnancy Register were retrospectively validated through standardized reviews of electronic medical records. The gold standard for classification followed the Swedish guidelines from the Swedish Society of Obstetrics and Gynecology (SFOG), updated in 2019. Incidence, sensitivity, and positive predictive value (PPV) were calculated and reported with 95% confidence intervals (CIs), using the Wilson score method. In addition, the study includes a descriptive analysis of preeclampsia disease characteristics and clinical diagnostic practices.

**Results:**

Among 7443 women included in the validation study, 843 (11.3%, 95% CI 10.6–12.1) had a HDP diagnosis recorded in the Swedish Pregnancy Register and underwent validation. Within the study population, the register‐based incidence of preeclampsia was 5.0% (95% CI 4.5–5.5%), while the minimum validated incidence was 5.1% (95% CI 4.6–5.6). The positive predictive value (PPV) of register diagnoses was 79.0% (95% CI 74.5–82.8%) for preeclampsia, 72.8% (95% CI 68.2–76.9%) for gestational hypertension, and 49.3% (95% CI 38.3–60.4%) for chronic hypertension, yielding an overall PPV for any hypertensive disorder of pregnancy diagnosis of 73.4% (95% CI 70.3–76.3%). Among validated cases of preeclampsia, 78% had proteinuria and the remaining cases were classified as preeclampsia based on other organ dysfunctions.

**Conclusions:**

In this contemporary Swedish cohort, a diagnosis of preeclampsia recorded in the Swedish Pregnancy Register demonstrated good validity, comparable to that reported in previous Nordic register validation studies.

## Graphical abstract

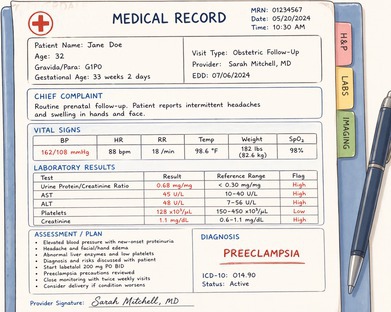

**Abbreviations**
CI: confidence interval
HDP/s: hypertensive disorder/s of pregnancy
HELLP syndrome: hemolysis, elevated liver enzymes and low platelet count syndrome
ICD‐10: the Swedish version of the International Classification of Diseases, 10th revision
IMPACT: study for Improving Maternal Pregnancy And Child ouTcomes
PPV: positive predictive value
SFOG: Swedish Society of Obstetrics and Gynecology
SGA: small for gestational age

**Key message**

In a structured validation of hypertensive disorders of pregnancy diagnoses in the Swedish Pregnancy Register, data from 7443 women revealed a 79.0% positive predictive value for preeclampsia, reflecting high diagnostic accuracy. Proteinuria was present in 78.0% of validated preeclampsia cases.

## 1 INTRODUCTION

The world Health Organization (WHO)'s systematic review of maternal deaths from 2009 to 2020, report hypertensive disorders as the second most common direct cause of death during pregnancy worldwide, even when excluding pre‐existing hypertensive disorders.^1^ Hypertensive disorders of pregnancy (HDP) affect 6–11% of all pregnancies and include preeclampsia, gestational hypertension and chronic hypertension. Preeclampsia, including HELLP syndrome (h*emolysis, elevated liver enzymes and* ^2^, ^3^, ^4^ *low platelets) is the most severe form that affects 2–8% of all pregnant women and can develop both de‐novo* as well as superimposed on underlying chronic hypertension.^3^, ^4^ Whilst chronic hypertension is not directly causally related to pregnancy, it is included in the HDPs as 20–50% of pregnant women with chronic hypertension develop superimposed preeclampsia,^5^ just as 15 to 25% of women with gestational hypertension progress to preeclampsia.^6^

In Sweden, the classification criteria for HDPs were updated in October 2019 by the Swedish Society of Obstetrics and Gynecology (SFOG), with criteria for organ dysfunction as shown in Table 1.^7^ The new definition of preeclampsia is more inclusive than the previous, primarily because significant proteinuria is no longer mandatory, and the threshold for defining proteinuria has been decreased. This broader definition better reflects the syndromic nature of preeclampsia with its various clinical presentations. The revision of the Swedish classification guidelines aligns with similar previous updates to international guidelines, such as those from the Danish Society for Obstetrics and Gynecology (DSOG), the American College of Obstetricians and Gynecologists (ACOG), and the International Society for the Study of Hypertension in Pregnancy (ISSHP).^2^, ^8^, ^9^

**TABLE 1:** Criteria for hypertensive disorders of pregnancy according to the 2019 Swedish Society of Obstetrics and Gynecology (SFOG) guidelines and their modifications in the study's validation scheme.

Diagnosis	SFOG classification criteria	Modifications in validation scheme*
Chronic hypertension	Diagnosis of hypertension or use of antihypertensive medication ≤20 gestational weeks.	Systolic BP ≥140 mmHg and diastolic BP ≥90 mmHg, measured twice ≥4 h apart. Antihypertensive medication present or initiated ≤20 gestational weeks.
Gestational hypertension	Hypertension onset ≥20 and normalization within 12 weeks postpartum. Systolic BP ≥140 mmHg and/or diastolic BP ≥90 mmHg, measured twice ≥15 min apart.	Also meets validation criteria if antihypertensive medication initiated at ≥20 gestational weeks or within 2 weeks postpartum.
Preeclampsia	Gestational hypertension plus at least one organ dysfunctions specified below. Proteinuria not mandatory.	
Superimposed preeclampsia	Chronic hypertension and at ≥20 gestational weeks: Persistent hypertension despite two antihypertensive medications, or at least one organ dysfunctions specified below. Proteinuria not mandatory.	
Eclampsia	Onset of seizures during pregnancy, labor, or within the first weeks postpartum without another cause (eg epilepsy). Proteinuria and hypertension not mandatory.	
HELLP syndrome	Hemolysis: haptoglobin (<0.25 g/L) or increased LD (>600 U/L or >10.0 μkat/L), thrombocytopenia (<100 × 10^9^/L) and elevated liver enzymes (AST ≥1.2 μkat/L or ALT ≥1.5 μkat/L). Proteinuria and hypertension not mandatory.	
Preeclampsia organ dysfunctions		
Proteinuria	Random urine albumin/creatinine ≥8 mg/mmol, or random urine protein/creatinine ≥30 mg/mmol, or urine albumin or protein ≥300 mg/24 h	Also meets validation criteria if urine dipstick ≥2+ once or 1+ twice consecutively
Other renal dysfunction		
Elevated serum creatinine	>90 umol/L	
Oliguria	<500 mL urine/24 h	
Kidney failure	Included but not defined	ICD‐10 code N17 in medical records
Hepatic dysfunction		
Elevated liver enzymes	Serum AST or ALT ≥2× upper normal value (1.2 or 1.5 μkat/L)	
Upper abdominal pain	Severe, persistent pain in epigastric or right subcostal area	
Hematologic dysfunction		
Thrombocytopenia	Platelets <100 × 10^9^/L or rapidly decreasing	Rapidly decreasing platelets not included
Hemolysis	Serum haptoglobin <0.25 g/L, or serum LD >600 U/L or >10.0 μkat/L	
Neurologic dysfunction		
Neurologic impairment	Severe headache or severe visual disturbance	Headache or visual disturbance not included, instead validated via magnesium sulfate administration for preeclampsia prophylaxis
Stroke	Included but not defined	ICD‐10 codes I61–I64 in medical records
Circulatory dysfunction		
Pulmonary edema	Included but not defined	ICD‐10 code J8 in medical records
Uteroplacental dysfunction		
Intrauterine growth restriction	Included but not defined	Fetal birthweight <−2 SD

Abbreviations: ALT, alanine aminotransferase; AST, aspartate aminotransferase; BP, blood pressure; HELLP syndrome, hemolysis, elevated liver enzymes and low platelet count syndrome; ICD, the Swedish version of the International Classification of Diseases 10th revision; LD, lactate dehydrogenase; SD, standard deviation; SFOG, Swedish Society of Obstetrics and Gynecology.

* The validation scheme used the SFOG guidelines with modifications according to the table.

Previous Nordic validation studies have reported high specificity of national register‐based diagnoses of preeclampsia, but lower sensitivity and substantial misclassification.^10^, ^11^, ^12^ These issues can compromise the data quality and validity of such registries. Ensuring the validity of diagnoses in a register is of utmost importance due to the fundamental role national registries play in research and quality control.

HDPs have not been validated in a Swedish context since the 1990s,^12^ and never within the Swedish Pregnancy Register or following the 2019 revision of the Swedish classification guidelines, in which proteinuria ceased to be a mandatory criterion for preeclampsia diagnosis. The objective of this study was therefore to assess the validity of HDP diagnoses in the Swedish Pregnancy Register, using medical record reviews as the gold standard and applying the updated Swedish diagnostic criteria in a contemporary cohort. Additionally, the study includes a descriptive analysis of preeclampsia disease characteristics and clinical diagnostic practices, such as the proportion of preeclampsia cases with documented proteinuria and the diagnostic methods employed to assess it.

## 2 MATERIAL AND METHODS

This study is a part of the prospective multicenter cohort study for Improving Maternal Pregnancy And Child ouTcomes (IMPACT). The IMPACT study was conducted across 13 sites in eight health care regions in Sweden (Supporting Information Table S1). It was designed to investigate first trimester prediction models of adverse pregnancy outcomes within the Swedish setting.^13^ Women were invited to participate while attending a publicly financed first‐trimester ultrasound scan, conducted between 11 + 0 and 14 + 6 weeks' gestation, from November 2018 to December 2022, with deliveries recorded through September 2023. Currently, approximately 80% of pregnant women in Sweden attend such a scan,^14^ and only regions offering such examinations were included in the IMPACT study. Participants were enrolled after receiving both written and oral information and providing informed consent. Each woman could participate only once. Women were not eligible if they were younger than 18 years of age or language barriers persisted despite interpreter support. Written information was available in 11 languages.

Women were seen once, and pre‐defined data were prospectively collected through interviews, medical examinations, and blood sampling and recorded in an electronic case report form (eCRF) at inclusion. Additional early pregnancy data and outcomes were collected post‐delivery via linkage with the Swedish Pregnancy Register. This is a high‐quality national quality register with near complete coverage,^14^ using the unique personal identity number of both mothers and infants.^15^ Data are automatically transferred directly from electronic medical records including the Swedish version of the International Classification of Diseases 10th revision (ICD‐10) codes from antenatal, obstetric, and neonatal care for both mother and infant, assigned by the responsible physician.

### 2.1 Study population

All women in the IMPACT cohort with registered deliveries from 22 + 0 gestational weeks, between January 2021 and July 2023, were eligible for inclusion in the validation study. This timeframe was chosen to ensure that all pregnancies occurred after the implementation of the updated SFOG classification criteria for HDPs in October 2019.^7^ Women were excluded if they were included in the IMPACT cohort but subsequently delivered in a healthcare region without a local assessor for medical record validation, delivered in a region not participating in the IMPACT study, or if the delivery region was unknown.

### 2.2 Validation process

Within the study population, a manual review of the electronic delivery hospital records was performed for all women with an ICD‐10 code of any HDP recorded in the Swedish Pregnancy Register. The diagnoses and corresponding diagnostic codes were: chronic hypertension (O10, I10, I12, I15); superimposed preeclampsia (O11); gestational hypertension (O13); preeclampsia, including HELLP syndrome (O14); eclampsia (O15).

The validation was conducted by local assessors in the participating regions. Antenatal, delivery, and postnatal medical records were reviewed and data on pre‐defined variables relevant for diagnosing HDPs were entered into a standardized Microsoft Excel® 2016 (Microsoft corp., Redmond, USA) spreadsheet. All variables used for HDP validation were obtained from review of the medical records, except for fetal birthweight standard deviation and ICD 10‐codes for kidney failure, pulmonary edema, and stroke (N17, J8, and I61‐64), which were retrieved from the Swedish Pregnancy Register. In cases of multifetal pregnancy, the birthweight of the smallest infant was recorded.

The gold standard for validated diagnoses of preeclampsia, gestational hypertension, and chronic hypertension was based on a modified version of the 2019 SFOG guidelines.^7^ The criteria and modifications applied in the study's validation scheme are detailed in Table 1. The modifications mainly concerned two areas: the assessment of proteinuria and subjective signs and symptoms. Although urine dipstick analysis is not included in the 2019 SFOG guidelines, it was incorporated into the validation protocol since several delivery hospitals in Sweden do not routinely perform other quantitative urine protein measurements. The SFOG guidelines also recognize rapidly decreasing platelet counts, severe headache, and persistent visual impairment as markers of organ dysfunction. However, these signs were excluded from the validation protocol due to the difficulty in reliably assessing them through electronic medical record review. For the validation of preeclampsia, no distinction was made between subtypes, including severe or superimposed forms. A gold‐standard diagnosis of preeclampsia was fulfilled if hypertension was present at any time during pregnancy or the immediate postpartum period, along with at least one type of organ dysfunction as specified in Table 1.

Proteinuria was not mandatory, but when present defined as elevated urinary protein according to one of the following criteria: (1) albumin/creatinine ≥8 mg/mmol or protein/creatinine ≥30 mg/mmol, (2) total albumin/protein >300 mg/24 h, or (3) urine dipstick analysis for albumin indicating ≥2+ once or = 1+ on two consecutive tests. Other forms of organ dysfunction included renal, hepatic, hematologic, neurologic, circulatory, or uteroplacental dysfunction defined as small for gestational age (SGA) birth, based on 2 SD (2.3rd percentile) below estimated birthweight for gestational age and sex, according to the Swedish reference curve for fetal growth^16^ (Table 1).

Markers of more severe disease included use of eclampsia prophylaxis with magnesium sulfate, elevated serum creatinine (>90 umol/L), hemolysis (serum haptoglobin <0.25 g/L or serum lactate dehydrogenase >10.0 μkat/L) and HELLP syndrome.

The electronic medical records were also reviewed for other potential causes of organ dysfunction, and clinical judgment was applied when relevant comorbidities were present. For instance, elevated liver enzymes were evaluated in relation to possible intrahepatic cholestasis of pregnancy, and pre‐existing chronic kidney disease was considered when evaluating serum creatinine levels and proteinuria.

### 2.3 Statistical analyses

Descriptive analyses were conducted to assess the incidence, sensitivity, and positive predictive value (PPV) for the HDP diagnoses preeclampsia, gestational hypertension, and chronic hypertension recorded in the Swedish Pregnancy Register. Descriptive statistics are presented as counts and/or proportions.

The incidence of each HDP diagnosis in the Swedish Pregnancy Register was calculated as the proportion of women in the study population with a corresponding diagnosis recorded in the register. The incidence of medical record validated diagnoses was calculated as the proportion of confirmed diagnoses in the study population and represents a minimum estimate since validation was conducted only for women with a register‐recorded HDP diagnosis.

The PPV for each diagnosis was calculated as the proportion of register‐recorded diagnoses that were confirmed by validation. Sensitivity was calculated as the proportion of women with validated diagnoses who also had the corresponding diagnosis recorded in the Swedish Pregnancy Register.

To further characterize the clinical presentation of preeclampsia, a descriptive analysis of the types and combinations of organ dysfunction, assessed both individually and in combination with proteinuria, was conducted.

Proportions were reported with 95% confidence intervals (CIs), calculated using the Wilson score method. Statistical analyses were conducted using R Software, version 4.2.3 (R Foundation for Statistical Computing, Vienna, Austria), with the binom package (version 1.1–1.1) used to calculate confidence intervals. Microsoft Excel® 2016 (Microsoft Corporation, Redmond, USA) was used for data handling and supplementary computations.

## 3 RESULTS

The IMPACT cohort comprises 12 908 participants, of whom the 7823 women who delivered between January 2021 and September 2023 were eligible for inclusion in this validation study. A total of 380 of these women were excluded, as they subsequently delivered in a healthcare region without a local validation assessor (one out of eight participating regions, *n* = 215), in a region not participating in the IMPACT study (*n* = 142), or if the delivery region was unknown (*n* = 23), resulting in a study population of 7443 women. Among these, 843 (11.3%, 95% CI 10.6–12.1%) women had an HDP recorded in the Swedish Pregnancy Register. This included 371 (5.0%, 95% CI 4.5–5.5%) women with preeclampsia, 397 (5.3%, 95% CI 4.8–5.9%) women with gestational hypertension, and 75 (1.0%, 95% CI 0.8–1.3%) women with chronic hypertension (Table 2). These women underwent manual validation of their HDP diagnoses according to SFOG Guidelines (Table 1). All women had available electronic medical records. Among the 843 women with HDP diagnoses recorded in the Swedish Pregnancy Register, 381 (45.2%) were validated as having preeclampsia, 351 (41.6%) gestational hypertension, 56 (6.6%) chronic hypertension, and 55 (6.5%) were not confirmed to have a validated diagnosis of HDP. This corresponds to a minimum validated incidence of any HDP of 10.6% (95% CI 9.9–11.3%), including 5.1% (95% CI 4.6–5.6%) with preeclampsia, 4.7% (95% CI 4.3–5.2%) with gestational hypertension, and 0.8% (95% CI 0.6–1.0%) with chronic hypertension (Table 2).

**TABLE 2:** Validation of hypertensive disorder of pregnancy diagnoses in the Swedish Pregnancy Register (SPR) among women in the IMPACT cohort 2021–2023, using the 2019 Swedish Society of Obstetrics and Gynecology (SFOG) guidelines as the reference standard (*n* = 843).

Diagnosis	Recorded in SPR (*n*)	Incidence in SPR* (95% CI)	Confirmed in medical records (*n*)	Minimum validated incidence* (95% CI)	True positives† (*n*)	Sensitivity‡ (95% CI)	PPV (95% CI)
Chronic hypertension	75	1.0% (0.8, 1.3)	56	0.8% (0.6, 1.0)	37	66.1% (53.0, 77.1)	49.3% (38.3, 60.4)
Gestational hypertension	397	5.3% (4.8, 5.9)	351	4.7% (4.3, 5.2)	289	82.3% (78.0, 86.0)	72.8% (68.2, 76.9)
Preeclampsia	371	5.0% (4.5, 5.5)	381	5.1% (4.6, 5.6)	292	76.9% (72.4, 80.9)	79.0% (74.5, 82.8)
Any HDP §	843	11.3% (10.6, 12.1)	788	10.6% (9.9, 11.3)	619	78.6% (75.6, 81.3)	73.4% (70.3, 76.3)

*Note*: Values are presented as counts or percentages with 95% confidence intervals (CI), calculated using the Wilson score method. ICD‐10 codes used to identify hypertensive disorders of pregnancy in the SPR were: chronic hypertension (O10, I10, I12, I15), including superimposed preeclampsia (O11); gestational hypertension (O13); and preeclampsia, including HELLP syndrome and eclampsia (O14, O15).

Abbreviations: CI, confidence interval; HDP, hypertensive disorder of pregnancy; PPV, positive predictive value; SFOG, Swedish Society of Obstetrics and Gynecology; SPR, Swedish Pregnancy Register.

* Incidence was calculated as the proportion of the 7443 women included in the study with each hypertensive disorder of pregnancy according to medical record validation. Since validation was conducted only for the 843 women with any hypertensive disorder of pregnancy recorded in the Swedish Pregnancy Register, these estimates may underestimate the true incidence and should be interpreted as lower‐bound estimates.

^†^ True positives: Cases for which the diagnosis recorded in the Swedish Pregnancy Register was confirmed as correct based on medical record validation.

^‡^ Sensitivity calculated among individuals with any hypertensive disorder of pregnancy diagnosis recorded in the Swedish Pregnancy Register; women without a registered diagnosis were not included in the medical record validation.

^§^ Any HDP refers to women with any hypertensive disorder of pregnancy (chronic hypertension, gestational hypertension, or preeclampsia).

The overall PPV for HDPs recorded in the Swedish Pregnancy Register was 73.4% (95% CI 70.3–76.3%). By condition, the PPV was 79.0% (95% CI 74.5–82.8%) for preeclampsia, 72.8% (95% CI 68.2–76.9%) for gestational hypertension, and 49.3% (95% CI 38.3–60.4%) for chronic hypertension (Table 2). The sensitivity of HDP registration in the Swedish Pregnancy Register is presented overall and stratified by specific HDP conditions in Table 2.

Of the 381 women with a validated preeclampsia diagnosis, 297 (78.0%) exhibited proteinuria, including 196 (51.4%) with isolated proteinuria and 101 (26.5%) with proteinuria combined with another organ dysfunction (Table 3). The remaining 84 validated preeclampsia cases (22.0%) lacked proteinuria but were classified as preeclampsia based on other organ dysfunctions, with 69.0% (*n* = 58) of these having a single organ system dysfunction.

**TABLE 3:** Distribution of specific organ dysfunctions among women with a validated preeclampsia diagnosis (*n* = 381) in the IMPACT cohort 2021–2023.

Validated diagnosis of preeclampsia (*n* = 381)						
	Isolated proteinuria (*n* = 196, 51.4%)	Not isolated proteinuria* (*n* = 185, 48.6%)				
		Overall	Other organ dysfunction and proteinuria (*n* = 101, 26.5%)	Other organ dysfunction without proteinuria (*n* = 84, 22.0%)		
				Overall	Multiple organ dysfunctions (*n* = 26, 6.8%)	Single organ dysfunction (*n* = 58, 15.2%)
Confirmed proteinuria†	132 (67.3%)	86 (46.5%)	86 (85.1%)	‐	‐	‐
Suggested proteinuria‡	64 (32.7%)	15 (8.1%)	15 (14.9%)	‐	‐	‐
Elevated liver enzymes	‐	58 (31.4%)	35 (34.7%)	23 (27.4%)	12 (46.2%)	11 (19.0%)
Upper abdominal pain	‐	52 (28.1%)	27 (26.7%)	25 (29.8%)	13 (50.0%)	12 (20.7%)
Eclampsia prophylaxis with magnesium sulfate	‐	50 (27.0%)	33 (32.7%)	17 (20.2%)	9 (34.6%)	8 (13.8%)
SGA at birth	‐	47 (25.4%)	21 (20.8%)	26 (31.0%)	7 (26.9%)	19 (32.8%)
Elevated creatinine	‐	32 (17.3%)	21 (20.8%)	11 (13.1%)	7 (26.9%)	4 (6.9%)
Thrombocytopenia	‐	20 (10.8%)	13 (12.9%)	7 (8.3%)	<5	<5
Hemolysis	‐	14 (7.6%)	11 (10.9%)	<5	<5	<5
Oliguria	‐	7 (3.8%)	5 (5.0%)	<5	<5	0
HELLP syndrome	‐	6 (3.2%)	5 (5.0%)	<5	<5	0
Pulmonary edema	‐	<5	<5	<5	<5	0
Kidney failure or stroke	‐	0	0	0	0	0

*Note*: Classification of organ dysfunction followed the 2019 guidelines of the Swedish Society of Obstetrics and Gynecology (SFOG). Values are presented as counts and percentages within each column/subgroup.

Abbreviations: HELLP, hemolysis, elevated liver enzymes, and low platelet count; SGA, small for gestational age; SFOG, Swedish Society of Obstetrics and Gynecology.

* Includes all women with at least one organ dysfunction other than proteinuria, regardless of the presence of proteinuria. Women with multiple organ dysfunctions are represented in more than one row.

^†^ Confirmed proteinuria was defined as a spot urine albumin/creatinine ≥8 mg/mmol, spot urine protein/creatinine ≥30 mg/mmol, or urine albumin or protein ≥300 mg in a 24‐h collection.

^‡^ Suggested proteinuria was defined as a urine dipstick analysis result of ≥2+ once or = 1+ on two consecutive occasions.

We compared women diagnosed with preeclampsia both according to the validation scheme and the Swedish Pregnancy Register (true positives, *n* = 292) with those who had preeclampsia according to the validation scheme but a diagnosis of gestational or chronic hypertension in the Swedish Pregnancy Register (false negatives, *n* = 89). Among those with validated preeclampsia and isolated proteinuria, a higher proportion of true positives had quantitative urine protein measurements rather than only dipstick analysis, compared with false negatives (81.0% vs. 26.5%). Additionally, a lower proportion of true positives had preeclampsia defined by organ dysfunction without proteinuria, compared to false negatives (17.5% vs. 37.1%).

Among women with a validated diagnosis of preeclampsia with both proteinuria and other organ dysfunction, the most common features were elevated liver enzymes, upper abdominal pain, eclampsia prophylaxis with magnesium sulfate, and infants born SGA.

Among the 58 women with validated preeclampsia without proteinuria and a single organ dysfunction, the most common manifestations were delivery of an SGA infant (32.8%), followed by upper abdominal pain (20.7%), and elevated liver enzymes (19.0%). (Table 3).

## 4 DISCUSSION

This study assessed the validity of HDP diagnoses among 843 women with such a diagnosis recorded in the Swedish Pregnancy Register, within a cohort of 7443 participants in the IMPACT cohort, yielding an overall PPV of 73.4% (95% CI 70.3–76.3%), and 79.0% (95% CI 74.5–82.8%) for preeclampsia. The incidence of preeclampsia was similar between register‐based and validated diagnoses (minimum limit of incidence) (5.0%, 95% CI 4.5–5.5%, and 5.1%, 95% CI 4.6–5.6%, respectively), and proteinuria was observed in 78% of validated diagnoses.

This is the first validation study of HDPs in a Swedish register since 1998,^12^ the first to specifically assess the Swedish Pregnancy Register, and the first to assess the updated, broader definition of preeclampsia introduced in Sweden 2019. The PPV of 79.0% (95% CI 74.5–82.8%) for preeclampsia aligns with findings from other recent Nordic studies using similar diagnostic criteria and registry data, including a Norwegian study from 2014 (83.9%),^11^ and a Danish study from 2016 (80.5%).^10^ However, the PPV observed in our study is lower than reported in the 1998 validation of preeclampsia diagnoses, based on ICD‐9 codes in the Swedish Medical Birth Register, which reported a PPV of 93%.^12^ Notably, both the Swedish Medical Birth Register^17^ and the Swedish Pregnancy Register^12^ obtain ICD codes directly from medical records.

The lower PPV observed in recent Nordic validation studies,^10^, ^11^ may reflect recent changes in both Swedish and international diagnostic guidelines. In particular, proteinuria is no longer required for diagnosis in the presence of other organ dysfunction, and the threshold for defining proteinuria has been reduced.^2^, ^7^, ^8^, ^9^ This aligns with our finding that a validated diagnosis of preeclampsia without proteinuria was more common among women without a register‐recorded diagnosis (false negatives) compared to those diagnosed both through the validation process and in the register (true positives). The new guidelines' broader inclusion criteria and increased complexity could lead to misclassification of preeclampsia without proteinuria to gestational or chronic hypertension instead. This misclassification could gradually decrease as the updated guidelines become more widely established in clinical practice. The lower‐limit estimate of the validated incidence of preeclampsia among women with HDP overall was slightly higher than the incidence reported in the register (5.1% vs. 5.0%, not statistically significant), while the incidence of gestational hypertension was lower (4.7% vs. 5.3%, not statistically significant), further suggesting a tendency to misclassify preeclampsia as less severe hypertensive conditions. This reasoning also aligns with the higher PPVs reported for ICD‐coded diagnoses of more clearly defined pregnancy‐related conditions, such as Down Syndrome (90.2%)^18^ and preterm birth (95.2%).^19^

For gestational hypertension, the PPV was 72.8% (95% CI 68.2–76.9%), notably higher than the 29.0% reported in the previously cited Danish study from 2016,^10^ but more comparable to the 56% found in an earlier Danish study from 2007.^20^ However, both Danish studies included relatively few gestational hypertension cases in the register (62 and 16 cases, respectively), which may limit the reliability and generalizability of their findings.

Among the HDP diagnoses, chronic hypertension demonstrated the lowest PPV, at 49.3% (95% CI 38.3–60.4%). No studies based on Nordic registers were identified for comparison. However, an American single‐center study using hospital discharge billing records in a high‐burden population (with a 37% prevalence of HDP) reported a PPV for chronic hypertension of 89.2%.^21^ The lack of validation studies of chronic hypertension in pregnancy‐related registers may reflect inherent challenges in accurately identifying this condition. These challenges likely include its relatively low incidence compared with other HDPs, the fact that it is often diagnosed by healthcare providers prior to pregnancy, and a substantial risk of misclassification due to difficulties in distinguishing it from gestational hypertension.

In the descriptive analysis of organ dysfunction among women with validated preeclampsia, markers of more severe disease were more common in cases with both proteinuria and other organ dysfunction than in those without proteinuria. Although the differences were not statistically significant, these findings generate several hypotheses for future research. They may partly reflect a higher rate of misclassification in the group without proteinuria due to reliance on less specific markers of preeclampsia, such as SGA, upper abdominal pain, and elevated liver enzymes, which can result from other underlying conditions. Specifically, SGA was the most common marker among women with a single organ dysfunction without proteinuria, highlighting the risk of misclassification when SGA alone is used to diagnose preeclampsia in hypertensive pregnancies, as only approximately half of SGA infants are truly growth‐restricted due to impaired uteroplacental dysfunction.^22^ Moreover, while SGA used for validation in this study was based on birth weight, preeclampsia diagnoses are typically coded during pregnancy when only ultrasound‐estimated fetal weight is available.

The main strength of this study is the large number of validated HDPs diagnoses in the Swedish Pregnancy Register, based on a study population across eight healthcare regions in Sweden, with narrow exclusion criteria and a structured validation approach. This supports the robustness and potential generalizability of the PPV estimates to the broader Swedish pregnant population.

Further strengths include that this is the first Swedish validation of ICD‐10‐based HDP diagnoses and the first validation study of any Swedish register following major revisions of the national preeclampsia criteria. As the Swedish Pregnancy Register automatically retrieves diagnostic codes from electronic medical records, validation of register‐based diagnoses reflects the accuracy of clinicians' diagnostic coding.

This study has some limitations. The IMPACT cohort comprises women who were invited to participate during a first‐trimester ultrasound scan, and who consented to participation, which introduces a risk of selection bias. However, approximately 80% of pregnant women in Sweden currently attend such scans.

Notably, certain indicators of organ dysfunction, such as decreasing platelet counts, severe headache, and persistent visual disturbances, were not included in the validation scheme, despite being part of current Swedish guidelines. These symptoms were excluded due to their subjective nature, while upper abdominal pain was included, as it was considered sufficiently specific when documented in electronic medical records. Consequently, the actual number of preeclampsia cases may have been slightly underestimated. However, this underestimation is likely partly counteracted by the inclusion of eclampsia prophylaxis with magnesium sulfate, since its indication according to the Swedish guidelines encompasses severe neurological symptoms. Moreover, defining proteinuria based on repeated 1+ protein on urine dipstick analysis may have led to an overestimation of proteinuria and consequently an overestimation of preeclampsia.

Furthermore, some cases of HDP according to gold standard in the cohort have probably been missed, as only women with a register‐recorded HDP diagnosis and not all deliveries underwent manual validation. Therefore, the reported sensitivity of validated HDP diagnoses likely represents an upper limit, and the validated incidence a lower limit. However, given the generally greater severity of preeclampsia, individuals fulfilling gold‐standard preeclampsia were likely included among the validated population of women with any of the HDPs. Therefore, the sensitivity and validated incidence estimate for preeclampsia are probably the most accurate among the three HDP diagnoses. Future studies of the Swedish Pregnancy Register that also include women without any HDP diagnosis would be valuable for estimating the negative predictive value and, more accurately, the sensitivity of register‐based diagnoses.

## 5 CONCLUSION

About four out of five register‐recorded preeclampsia diagnoses were correctly classified according to national guidelines, with a similarly high upper‐limit sensitivity. Further, proteinuria was present in 78.0% of all validated preeclampsia cases. The PPV and sensitivity were lower for gestational hypertension and chronic hypertension, suggesting that many women with HDPs may receive suboptimal clinical care and follow‐up. These findings highlight the clinical need for improved diagnostic procedures. Furthermore, researchers should account for this misclassification when using register‐based diagnoses.

## AUTHOR CONTRIBUTIONS

AS, LB, YC, SH, SRH, PC, PL, MK, AL, and AKW contributed to the conceptualization of the study and to funding acquisition. MF, AS, LB, YC, and AKW planned the study design. MF and HI performed the statistical analyses. MF and AS interpreted the data, and MF drafted the first version of the manuscript with support from AS. All authors reviewed and approved the final manuscript.

## FUNDING INFORMATION

Anna Sandström was funded by the Swedish Society of Medicine (AS FoUI‐971120). Lina Bergman was supported by the Wallenberg Centre for Molecular and Translational Medicine, Sahlgrenska University Hospital, and Gothenburg University. Ylva Carlsson and Anna Sandström were funded by grants from the Swedish state under the ALF agreement between the Swedish government and the county councils (ALFGBG‐75710, ALFGBG‐77860, ALFRS‐20190448, ALFRS‐20200735). The remaining authors received no specific funding for this project.

## CONFLICT OF INTEREST STATEMENT

Ylva Carlsson, Lina Bergman, Peter Conner, Stefan Hansson, Marius Kublickas, Peter Lindgren, Anders Larsson, Anna‐Karin Wikström, and Anna Sandström are members of the steering committee for the IMPACT study. Perkin Elmer, Roche Diagnostics, and Thermo Fisher provided reagents used in the IMPACT study for the analysis of placental growth factor (PlGF) free of charge. Perkin Elmer, Roche Diagnostics, and Thermo Fisher also contributed to running costs for analyses of placental growth factor. Written agreements are in place stating that the companies have no commercial rights to the results and no rights to the generated data, except access to their own PlGF results, and that they have no rights to deny publication of any data nor have the right to any publications concerning the project. Lina Bergman has consulted for DiaMedica and Araka for new therapeutics in preeclampsia and received drugs and placebo from Merck Damstadt for a treatment trial in preeclampsia. Henrik Imberg has served as a statistical consultant for DiaMedica. Mark Frick and Sara Hellström have no conflict of interest.

## ETHICS STATEMENT

The study was approved by the Regional Scientific Ethics Committee of Uppsala, initial application number 2018/231 (date July 4, 2018), amendment number 2018/231/1 (date November 6, 2018), and the Swedish Ethics Review Authority, amendment number 2020/00319 (date February 12, 2020) and amendment number 2022/05690/02 (date November 21, 2022).

## Supporting information


**Table S1.** Distribution of study participants by Swedish healthcare region of delivery in the IMPACT cohort, 2021–2023.

## Data Availability

The data that support the findings of this study are available on request from the corresponding author. The data are not publicly available due to privacy or ethical restrictions.
