# Supplementary material for: Validity of hypertensive disorders of pregnancy diagnoses in the Swedish pregnancy register using a contemporary cohort
Source: Acta Obstet Gynecol Scand. 2026 Jun 12;105(9):1800–9. doi: 10.1111/aogs.70286 (PMC13394963; doi:10.1111/aogs.70286)
Supplement: Supplementary file 1 [file AOGS-105-1800-s001.docx]

**Supporting information Table S1.** Distribution of study participants by Swedish healthcare region of delivery in the IMPACT cohort, 2021–2023.

| **Health-care region** | **Number of included women** |
| --- | --- |
| Dalarna | 1546 |
| Skåne | 52 |
| Stockholm | 1325 |
| Uppsala | 1429 |
| Värmland | 669 |
| Västra Götaland | 2193 |
| Örebro | 229 |
